# Active swimming and transport by currents observed in Japanese eels (*Anguilla japonica*) acoustically tracked in the western North Pacific

**DOI:** 10.1038/s41598-022-05880-x

**Published:** 2022-03-01

**Authors:** Nobuto Fukuda, Toshihiro Yamamoto, Kazuki Yokouchi, Hiroaki Kurogi, Makoto Okazaki, Yoichi Miyake, Tomowo Watanabe, Seinen Chow

**Affiliations:** 1 Japan Fisheries Research and Education Agency, Yokohama Station2-12-4 Fukuura, Kanazawa, Yokohama, Kanagawa 236-8648 Japan; 2 Japan Fisheries Research and Education Agency, Hatsukaichi Station2-17-5 Maruishi, Hatsukaichi, Hiroshima 739-0452 Japan

**Keywords:** Animal migration, Animal behaviour, Ichthyology

## Abstract

The mechanisms of oceanic animal migration remain enigmatic. Adult Japanese eels start their long-distance oceanic migration from coastal areas to breed near the West Mariana Ridge. We tracked acoustically tagged eels released in the Kuroshio Current (KC) area near Japan (five silver-phase eels, three of which had impaired swim bladders) and a tropical/subtropical (TS) area near/in the spawning area (two yellow-phase and three silver-phase eels). We analyzed their active swimming and transport by water currents. The strong flow of the KC dominated the eels’ movements in the north, and TS area; their swimming influenced their movements. In the KC area, greater distances were covered at night than during the day, because eels swam in shallower layers with strong currents at night. Three and one eel in the TS and KC area in the upper 400 m showed counterclockwise and clockwise movements around the time of solar culmination, respectively. The meta-analysis showed that eels released at middle latitudes (20°–34° N) generally swam southward through currents, whereas those released at low latitudes (12°–13° N) generally swam northward through currents. Our study suggests the influence of the surrounding current and a potential effect of solar cues on the movements of Japanese eels.

## Introduction

Temperate catadromous eels of the genus *Anguilla* are among the most enigmatic animals exhibiting a long-distance-oriented migration^1, 2^. Anguillid eels are born in the open ocean, their larvae grow drifting with oceanic currents^1, 3, 4^. Following this, they migrate to freshwater and estuarine growth habitats. They spend years in continental and coastal waters, and then they metamorphose into silver-phase eels and migrate toward their spawning areas^5–7^. Temperate anguillid eels migrate thousands of kilometers^8^, swimming primarily in the mesopelagic zone, showing diel vertical migration (DVM) swimming in relatively shallow water (mainly 150–250 m) during the night and deep water (mainly 500–800 m) during the day to reach their spawning sites^9–22^ without feeding^23^.

Pop-up satellite archival tags (PSATs) have become increasingly popular for collecting data on the water temperature, depth, and light intensity experienced by catadromous eels during oceanic migration^10–15, 17–21^. The migration route of the American eel, *Anguilla rostrata*, to the northern edge of the Sargasso Sea was partially determined using depth data transmitted from PSATs^17^. However, elucidating the migration routes of the eels does not resolve the navigation cues that orient their swimming, as it is not possible to distinguish active swimming from passive transport by currents using the PSAT data alone. Furthermore, satellite-based tracking relies on ocean circulation models^17, 18^, but the daily locations reconstructed from the PSAT data have high uncertainty with more than 100 km on average^17^. This uncertainty must cause difficulty in differentiating passive transport and active swimming.

Acoustic telemetry is a useful technique for tracking fish^24^ that has been used in several tracking studies on migrating anguillid eels^9, 16, 25–29^, which have reported swimming behaviors such as DVM, migration speed, and migration routes. If the water currents are measured at the same time, the fine-scale active swimming by fish can be determined using an analysis that considers the speed and direction of water currents, but these types of studies are limited because active tracking, which generally involves the tracking of only one individual at a time, is costly and laborious, especially in offshore areas. There were only a few studies in estuarine or coastal area attempting to investigate the active swimming behaviors of the American eel and European eel, *A. anguilla*, in relation to tidal currents using acoustic tags and a current meter^25, 26^. The fine-scale movement by active swimming and transport by current of anguillid eels have not been assessed in the open ocean.

Among the anguillid species, the Japanese eel is an ideal model for the investigation of mesopelagic navigation as its life cycle has been well investigated. This species spawns along the western side of the West Mariana Ridge. Unlike other catadromous eels, their spawning sites have been definitively identified through the collection of fertilized eggs, newly hatched larvae, and mature adults^30–36^. Thus, the departure and destination of their oceanic migration are well known. One of the general hypotheses for the migration route of the Japanese eel is that the eels departing from Japan are transported eastward in the KC and thereafter turn southward^2, 37^. The first PSAT study of Japanese eels demonstrated that they moved along the KC^14^; however, the drag caused by the external tag could affect their swimming performance^38–40^. It remains in question whether the hypothesized migration route has been traced without the effects of drag by the external tag.

Several laboratory experiments of anguillid eels have shown that magnetic cues may be used by juvenile and adult eels to navigate during migration^41–44^, and this appears to be true for temperate anguillid eels that likely use a magnetic sense for their spawning migrations^45^. Whereas a recent comprehensive review indicated that no single cue or mechanism is used by animals to enable successful navigation over thousands of kilometers^46^. Among terrestrial animals, the use of celestial navigational cues is fairly widespread^47–51^. Generally, aquatic animals that inhabit deeper layers, such as the mesopelagic zone (200–1000 m), are unlikely to use celestial cues because the quality of information from the light of celestial bodies, including information on the refracted angle and intensity of light, declines as water depth increases. However, a polarization pattern, which could be used to locate the Sun’s azimuth, was observed down to at a depth of 200 m at the minimum in clear water, possibly extending to over 1000 m in more transparent waters^52, 53^. Thus, such fainter cues could potentially be used for migratory navigation.

In 2010 and 2012, we performed acoustic telemetry tracking of Japanese eels in the tropical–subtropical (TS) areas near/in their spawning area and the Kuroshio Current (KC) area off the coast of Japan. The DVM of wild-caught eels corresponding to sunrise and sunset has previously been reported using these tracking data^16^; however, the horizontal movements of these eels have not been analyzed. The North Equatorial Current in the spawning area has a westward flow of ~ 15–30 cm/s near the surface^54^, while the KC, i.e., the western boundary current of the North Pacific Ocean, has a strong northeastward flow that can reach speeds > 100 cm/s near the surface^55^. When combined with environmental water flow data measured using an Acoustic Doppler Current Profiler (ADCP) system, fine-scale vectors of movements through currents (called “active swimming” or “swimming” vectors in this study) can be calculated by subtracting the water-current vectors from the fish travel vectors. The tracked eels in this study had various attributes that included two yellow-phase and three silver-phase eels in the TS area as well as five silver-phase eels in the KC, of which three eels had impaired swim bladders. Here, we preliminarily analyzed horizontal-swimming characteristics such as active swimming and transport by currents of these eels in the open ocean, and their mysterious oceanic migration was discussed in relation to the surrounding current and potential solar cues.

## Results

### Eel swimming speed in different environments

To analyze the active swimming (excluding current transport) and transport by current of the eels, we defined several categories of types of analyses. The horizontal movement of fish (including both swimming and current transport) was defined as a travel, and the length of the trajectory indicated a travel distance. The swimming and current vectors were obtained at 10-min intervals. The magnitudes of each were defined as swimming speed and transport speed, respectively, which represents the speeds of each when viewed in short time. The mean vectors of 10-min-interval swimming vectors during day and night over the tracking periods were calculated to measure the average velocity by active swimming over each period, i.e., the net displacement divided by the time traveled, in which the magnitude of the mean vector was defined as effective swimming speed.

**Figure 1:**
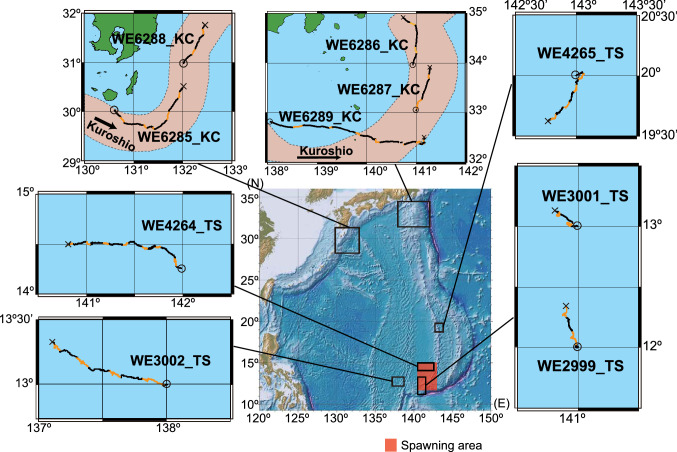
Track maps for ten eels used for analyses in this study. Trajectories of ship tracking ten eels during daytime (yellow line) and nighttime (black line). Start (○) and end points (×) of tracking. The topographic and bathymetric map (central bottom) were created using Generic Mapping Tools ver.4 (https://www.generic-mapping-tools.org) and the database ETOPO1 grid (https://www.ngdc.noaa.gov) was used. Other maps were drawn using QGIS software (https://qgis.org) and the database GSHHG ver. 2.2.2 (http://www.soest.hawaii.edu). These maps were further modified using Adobe illustrator ver. 24. The Kuroshio Current (orange area) is drawn based on the data from the Quick Bulletin of Ocean Conditions by the Japan Coast Guard’s Hydrographic and Oceanographic Department, collected on 28 November and 16 December 2012 (http://www1.kaiho.mlit.go.jp). The spawning area is predicted by collecting fertilized eggs, newly hatched larvae and matured adults is shaded in red.

Five (two yellow-phase and three silver-phase) eels were tracked in the TS area from July to August 2010, and five silver-phase eels were tracked in the KC area from November to December 2012 (Fig. 1). The tracking period and travel distance for each eel ranged from 42.5 to 163.4 h and 56.1 to 217.8 km, respectively, in the TS area and 38.2 to 191.0 h and 106.0 to 379.9 km, respectively, in the KC area (Table 1). The travel distances and trajectories during the night tended to be longer and more linear than during the day (Fig. 1). Moreover, the travel distance per day was significantly larger in the KC area (mean ± SD: 50.9 ± 16.9 km/day, i.e., 59 ± 20 cm/s, n = 14) than in the TS area (31.9 ± 4.5 km/day, i.e., 37 ± 5 cm/s, n = 14) (Welch’s t-test, *P* < 0.05).

**Table 1:** Summary of tracking experiments for 11 Japanese eels (*Anguilla japonica*). KC: Kuroshio Current area, TS: Tropical-subtropical area.

ID^1^	Release location	Origin	TL (cm)	BW (g)	EI^2^	Stage^3^	Released at				Tracking ceased at				Period (h)	Travel distance (km)
							Date	Time	Lat (N)	Long (E)	Date	Time	Lat (N)	Long (E)		
WE6285_KC	KC	Amikake River, Kagoshima, November 2012	72.6	702	5.97	S1	28-Nov-2012	21:27	30º 02′	130º 36′	3-Dec-2012	21:30	30º 29′	131º 59′	118.0	256.1
WE6288_KC^4^	KC	Naka River, Tokyo, November 2012	83.4	996	4.95	S2	4-Dec-2012	21:13	30º 58′	132º 01′	6-Dec-2012	21:13	31º 45′	132º 27′	48.0	106.0
WE6289_KC	KC	Naka River, Tokyo, November 2012	79.6	902	5.54	S2	7-Dec-2012	22:42	32º 49′	138º 03′	15-Dec-2012	22:42	32º 30′	141º 09′	191.0	379.9
WE6287_KC^4^	KC	Naka River, Tokyo, November 2012	81.8	914	4.67	S2	16-Dec-2012	05:19	33º 03'	140º59'	17-Dec-2012	20:00	33º54'	141º18'	38.2	108.3
WE6286_KC^4^	KC	Naka River, Tokyo, November 2012	83.4	990	5.95	S2	17-Dec-2012	23:17	33º57'	140º56'	19-Dec-2012	23:17	34º55'	140º44'	48.0	125.9
WE2999_TS	TS	Tone River, Chiba, September 2009	79.6	860	4.17	S1	10-Jul-2010	23:30	12º00′	140º 59′	12-Jul-2010	18:35	12º 20′	140º 54′	43.1	56.9
WE3001_TS	TS	Tone River, Chiba, September 2009	77.5	740	5.11	S1	13-Jul-2010	00:25	13º 00′	140º 59′	14-Jul-2010	19:10	13º 08′	140º 49′	42.5	56.1
WE3002_TS	TS	Tone River, Chiba, September 2009	90.1	1000	3.97	S1	16-Jul-2010	21:10	13º 00′	138º 00′	20-Jul-2010	21:00	13º 16′	137º 18′	95.8	132.4
WE4263_TS^6^	TS	Tone River, Chiba, September 2009	70.5	660	5.46	Y2^5^	24-Jul-2010	01:10	13º 00′	137º 59′	26-Jul-2010	20:00	13º 11′	137º 39′	67.3	88.8
WE4264_TS	TS	Tone River, Chiba, September 2009	77.5	580	5.70	Y2^5^	8-Aug-2010	00:35	14º 15′	141º 59′	14-Aug-2010	20:00	14º 29′	140º 49′	163.4	217.8
WE4265_TS	TS	Tone River, Chiba, September 2009	72.2	460	4.96	Y2^5^	15-Aug-2010	20:18	20º 00′	143º 00′	19-Aug-2010	22:10	19º 37′	142º 46′	97.9	108.6

^1^It consists of the ID in the study by Chow et al.^16^ plus the release location. ^2^Eye index (= {[(A + B)/4]^2^ × π/TL(mm)} × 100, A; horizontal orbital diameter (mm), B; vertical orbital diameter (mm)). ^3^Stages were determined from the the colorations of pectoral fins and ventral skin according to Okamura et al.^66^. ^4^Swimbladders of these wild eels were surgically malfunctioned. ^5^They were Y2 stage from a view of body colorations but had eye index ranging in the S1 stage in Okamura et al.^66^. ^6^The eel (WE4263_TS) did not exhibit normal diel vertical migration, so this eel was exclueded from the horizontal swimming analysis.

In the KC area, the average swimming speeds, i.e., the average distance of 10-min interval swimming vectors per second in this study, of each eel were 26–40 cm/s during the day (mean depths: 393–608 m) and 34–40 cm/s during the night (mean depths: 139–281 m) (Table 2). The average transport speeds, i.e., the average distance of 10-min-interval current vectors of eel swimming depths per second, were much higher in the shallow layer where the eels swam during the night (65–103 cm/s) than those in the deep layer where they swam during the day (27–58 cm/s). The swimming speeds in body length per second were significantly higher during the night (mean ± SD, 0.48 ± 0.29 BL/s) than during the day (mean ± SD, 0.41 ± 0.27 BL/s) and were not significantly influenced by swim bladder impairment, which was used by Chow et al.^16^ to investigate the effect of the swim bladder on vertical movement (Table 3).

**Table 2:** Summary of swimming and transport vectors obtained at 10-min intervals in the acoustic tracking of ten Japanese eels. Values in parenthesis indicate standard deviations. Asterisk(*) in the bearing of the swimming vector indicates a significant difference from the uniformity of a circular distribution by the Rayleigh test (*P* < 0.05).

Release area	Time zone	Eel ID	N	Swimming vector at 10-min intervals		Transport vector at 10-min intervals		Swimmning velocity over tracking periods	
				Mean magnitude (cm/s)	Mean bearing (º)	Mean magnitude (cm/s)	Mean bearing (º)	Magnitude (cm/s)	Bearing (º)
Kuroshio Current	Daytime	WE6285_KC	280	32 (22)	155º (1.92)*	27 (14)	47º (1.49)	6	159
		WE6288_KC	115	26 (16)	254º (1.92)	36 (5)	25º (0.17)	7	261
		WE6289_KC	384	33 (23)	199º (1.57)*	28 (20)	67º (1.03)	7	200
		WE6287_KC	107	40 (19)	185º (1.64)*	58 (29)	13º (0.26)	10	189
		WE6286_KC	103	29 (17)	221º (1.23)*	44 (20)	339º (0.48)	16	217
	Nighttime	WE6285_KC	286	40 (20)	120º (1.31)*	65 (26)	44º (0.56)	18	116
		WE6288_KC	85	34 (20)	236º (1.83)	83 (11)	29º (0.12)	7	218
		WE6289_KC	496	37 (25)	205º (1.34)*	72 (48)	70º (0.58)	16	212
		WE6287_KC	84	38 (19)	304º (1.50)*	103 (14)	23º (0.14)	15	309
		WE6286_KC	94	34 (22)	271º (1.98)	89 (39)	332º (0.37)	8	270
Tropical-subtropical	Daytime	WE4265_TS	291	29 (18)	226º (1.49)*	9 (5)	233º (1.56)	10	220
		WE4264_TS	504	31 (20)	271º (1.94)*	10 (6)	298º (0.85)	3	268
		WE3001_TS	146	36 (17)	265º (1.96)*	12 (5)	67º (0.96)	5	265
		WE2999_TS	146	38 (15)	342º (1.00)*	10 (6)	86º (1.84)	23	344
		WE3002_TS	292	39 (16)	310º (0.97)*	12 (7)	177º (1.22)	24	312
	Nighttime	WE4265_TS	188	29 (17)	226º (1.53)*	13 (6)	193º (1.06)	10	209
		WE4264_TS	320	35 (09)	290º (1.48)*	29 (9)	263º (0.52)	11	283
		WE3001_TS	57	35 (15)	308º (0.82)*	12 (6)	290º (0.73)	26	310
		WE2999_TS	53	38 (15)	348º (0.75)*	11 (5)	290º (1.11)	30	355
		WE3002_TS	168	41 (16)	292º (0.85)*	13 (9)	249º (0.94)	29	291

**Table 3:** Summary of linear mixed models fitted for swimming speeds in body length per second.

	Estimate	Std. error	df	t	*p*
(Intercept)	0.433	0.040	2.76	10.83	0.002**
Time of day: Night	0.070	0.012	2028.52	5.64	< 0.001***
Swimbladder treatment: Intact	− 0.067	0.052	2.80	− 1.30	0.292
(Intercept)	0.448	0.028	4.32	15.85	< 0.001***
Time of day: Night	0.036	0.010	2466.00	3.76	< 0.001***
Developmental stage: Yellow-phase	− 0.012	0.039	4.06	− 0.30	0.780

Signif. codes: 0 ‘***’ 0.001 ‘**’ 0.01 ‘*’ 0.05 ‘.’ 0.1 ‘ ’ 1.

In the TS area, the average swimming speeds of each eel were 29–39 cm/s during the day (mean depths: 353–538 m) and 29–41 cm/s during the night (mean depths: 162–242 m) (Table 2). The average transport speeds in the deeper layer where they swam during the day were lower (9–12 cm/s) than in the shallower layer where they swam during the night (11–29 cm/s). Moreover, the swimming speeds in body length per second were significantly higher during the night (mean ± SD, 0.46 ± 0.23 BL/s) than during the day (mean ± SD, 0.43 ± 0.23 BL/s) and were not significantly different between the yellow-phase and silver-phase eels (Table 3).

The effective swimming speed during day and night over the tracking periods were lower than the abovementioned short-term swimming speeds as the swimming vectors at the 10-min intervals exhibited more or less directional variation (Table 2). The effective swimming speed of each eel were 6–16 cm/s during the day and 7–18 cm/s during the night in the KC area and 3–24 cm/s during the day and 10–30 cm/s during the night in the TS area (Table 2).

### Eel swimming direction

In the KC area, the mean direction of the 10-min interval swimming vectors of each individual was eastward (mean bearing: 120°) to westward (304°), whereas the mean direction of the 10-min interval transport vectors was northward (339°) to eastward (70°) (Table 2). In the TS area, the mean direction of the 10-min interval swimming vectors of each individual was southwestward (226°) to northward (348°), whereas the mean direction of the 10-min interval transport vectors was northeastward (67°) to westward (298°) (Table 2). All five eels in the TS area exhibited a directional swimming during the day and night, whereas in the KC area, four of the five eels exhibited a directional swimming during the day and three of the five eels during the night (Rayleigh test, *P* < 0.05, Table 2).

**Figure 2:**
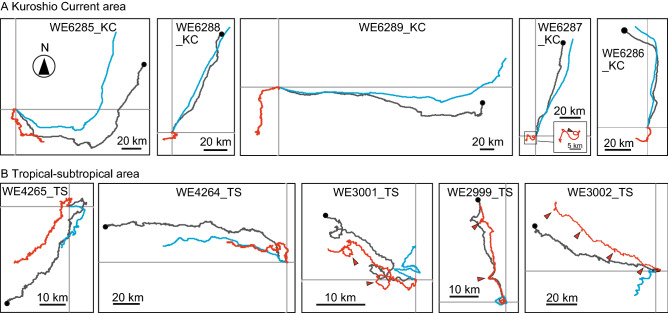
Trajectories during tracking eels. Travel trajectories are shown by gray lines, swimming trajectories by red lines and transport trajectories by blue lines. Vertical and horizontal lines indicate north–south axis and east–west axis with north at the top. The crossing points of two lines indicate release points. The closed circles indicate end points of tracking. Circular or bending curves around the time of solar culmination are shown by red triangles.

The swimming and transport trajectories are superimposed with the travel trajectories in Fig. 2. These trajectories were drawn by the progressive vector plots of the eel’s active swimming through the water, transport by currents and resulting eel movement over the ground. In the KC area, the transport trajectories (Fig. 2A) were similar to the travel trajectories. This indicates that the flow of the KC largely dominated the movements of the eels and the swimming by eels had a little effect on the travel trajectories. Contrarily, the swimming trajectories in the TS area (Fig. 2B) were similar to the travel trajectories, indicating that the swimming by eels significantly contributes to the travel.

**Figure 3:**
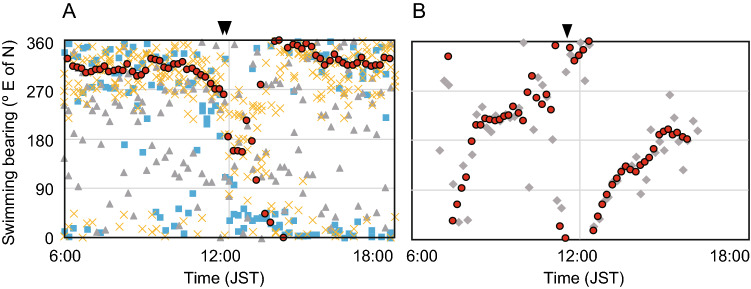
Time-series fluctuation of swimming bearing of four eels. (**A**) The fluctuations of swimming bearings in three eels (gray triangle WE2999_TS, blue square WE3001_TS, yellow cross WE3002_TS) released at 12°–13°N in the tropical–subtropical area around the time of Sun culmination (inverted black triangles). (**B**) The fluctuations of swimming bearings in one eel (gray diamond WE6287_KC) released at 33°N in the Kuroshio Current area changed swimming bearings around the time of solar culmination. Red circles in both figures indicate the 50 min moving average of swimming bearings.

**Figure 4:**
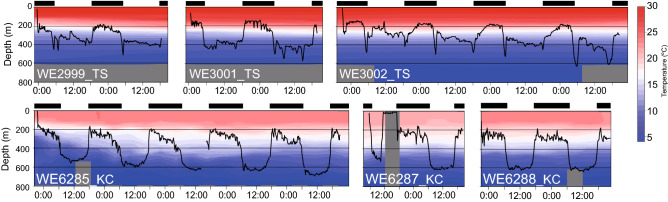
Vertical migration behavior of six eels and temperature structures. The swimming depths of three eels (WE2999_TS, WE3001_TS, WE3002_TS) released in the tropical–subtropical area and three eels (WE6285_KC, WE6287_KC, WE6288_KC) released in the Kuroshio Current area were written by black lines. The vertical profiles of temperatures in the eels’ swimming place were drawn using color coded from blue (5 °C) to red (30 °C). No temperature data was available for gray area. Black bars indicate nighttime.

Around the time of solar culmination when the Sun reaches its highest point above the horizon each day, circular or bending curves were observed in the swimming trajectories of several eels (Fig. 2, red arrowhead). Counterclockwise and bending curves were observed around the time of solar culmination in three eels (WE2999_TS, WE3001_TS, and WE3002_TS) released at low latitudes (12–13°N in the TS area), where the Sun passed from east to west in the northern sky. Figure 3A presents the time-series changes in the swimming directions of eels, indicating that they moved in a southerly direction when the Sun culminated in the northern sky. These three eels first dove deeper around sunrise and came up shallower and then swam at depths of approximately 300–400 m during the day (Fig. 4). Clockwise movement by eels was observed on 1 of 22 days (the first day of WE6287_KC) at middle latitudes (north of 20° N), where the Sun passed from east to west in the southern sky. This eel was swimming in an irregular manner in very shallow water during the first day (Fig. 4). The swimming directions of this eel indicate that it moved in a northerly direction when the Sun culminated in the southern sky (Fig. 3B).

**Figure 5:**
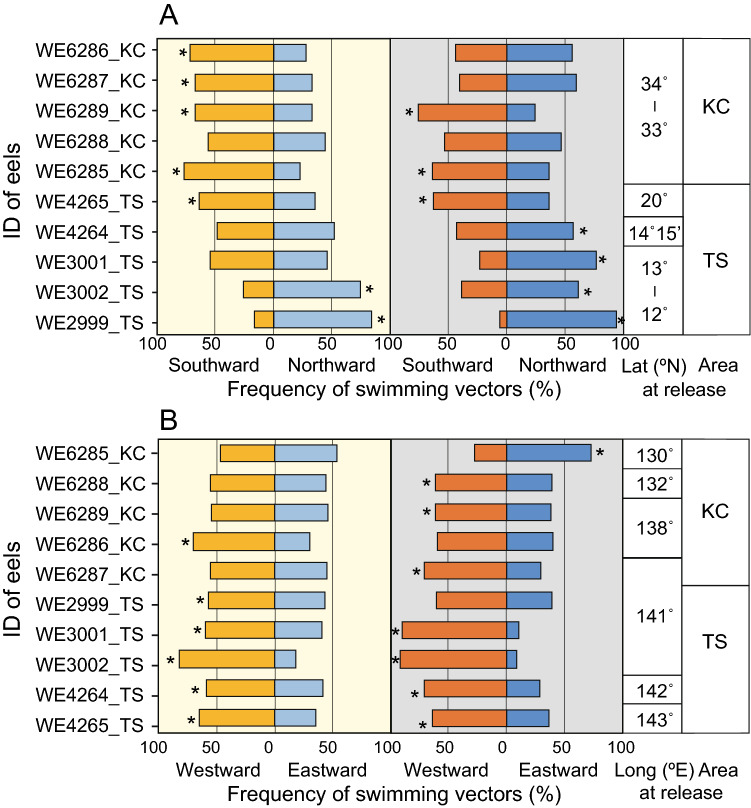
Frequencies of swimming directions of tracked eels in meridional and zonal axis. (**A**) Frequency of northward and southward swimming vectors during daytime (yellow shading) and nighttime (gray shading). Vectors with the bearing of 0°–90° and 270°–360° are categorized as northward, while those at 90°–270° are southward. Eels released at higher latitudes are ordered from the top. The latitude and area of release points are shown in the right box. B. Frequency of eastward and westward swimming vectors during daytime and nighttime. Vectors with the bearing of 0°–180° and 180°–360° are eastward and westward, respectively. Eels released at more west positions are ordered from the top. The longitude and area of release points are shown in the right box. Asterisks (*) indicate a significant difference in comparison with a uniform expected frequency under the null hypothesis by Chi-square test (*P* < 0.05).

A meta-analysis among the ten tracked individuals was performed to examine whether the swimming along the north–south and east–west axis was associated with the latitudinal and longitudinal positions of the tracking area. In the meridional axis (north–south), the swimming trajectories of eels released in the KC area showed that they swam southward from the release points (Fig. 2A), whereas those of eels released in the TS area, except WE4265_TS (released at 20°N), demonstrate that they swam northward from the release points (Fig. 2B). The 10-min interval swimming vectors of eels released at 12° to 13° N tended to have northward components (0–90° and 270–360°) with significantly higher frequencies, whereas the swimming vectors of eels released at 20° to 34° N tended to have southward components (90°–270°) with significantly higher frequencies (Fig. 5A).

In the zonal axis (east–west), swimming vectors with westward components (180°–360°) were more frequently observed in eels released at the TS area than in eels at the KC area (Fig. 5B). Their tendency to swim in an east–west direction did not depend on the longitudinal position of the release points toward the spawning area. In addition, no notable difference was observed in these meridional and zonal movements between day and night.

## Discussion

To our knowledge, this study provides the first recorded information on the active swimming of Japanese eels and on their transport by currents in the open ocean. Specifically, the strong flow of the KC largely dominated the movements of the eels and transported them northeastward while they swam mainly southward, and active swimming contributed a little to their travel trajectories. In contrast, the swimming of eels made a relatively higher contribution to their travel trajectories in the TS area.

Our in situ estimates of the mean swimming speeds of Japanese eels (26–41 cm/s) were similar or slightly lower than those of European eels. In the acoustic tracking experiment of European eels considering environmental current vectors, their swimming speeds were 35–58 cm/s in the coastal midwater^26^. In a laboratory experiment using stamina tunnels with stable temperatures, the optimal swimming speeds of European eels were estimated to be 61–68 cm/s (0.74–1.02 BL/s)^56^, which were higher than the in situ estimates. The minimum swimming speed of European eels is considered to be 40 cm/s if they will arrive at their spawning area in the Sargasso Sea (distance of 5500 km) in 6 months, and their optimal swimming speeds were sufficient to migrate over the long distance in time for the near-spawning period after escape from their growth habitats^56^. However, field studies using PSAT tagging also reported that in situ migration speeds (including transport by currents) were less than the optimal swimming speeds and suggested that some European eels could reach their spawning area within the near-spawning periods and that others only arrive in time for the following spawning season^19^.

Our estimated effective swimming speed of Japanese eels, all day and all night over the tracking periods, ranged from 3 to 30 cm/s with individual variations. These estimates were consistent with the swimming speeds (excluding transport by currents) of 2.2–15.1 km/day (2–18 cm/s) estimated in the PSAT study of Japanese eels^14^. Silver-phase Japanese eels start migrating from their coastal growth habitats in Japan primarily in October to December^57, 58^, and spawning near the West Mariana Ridge occurs in April to August^33, 35^. Numerical models assuming that migrating eels use true navigation (readjusted compass) or a constant compass heading (fixed compass from the departure place to the spawning site) indicate that the minimal swimming speed required to arrive at the spawning area within 8 months is 10–12 cm/s^37^. Our estimated effective swimming speeds of five out of ten eels during the day and eight out of ten eels during the night were similar or higher than these minimal speeds. The low effective swimming speeds frequently observed during the day might be due to the relatively low values observed in the swimming speed at 10 min intervals and the swimming directions often varying during the day. When eels swim with stable orientation, as observed in three of the eels (WE2999_TS, WE3001_TS, and WE3002_TS) during the night, the effective swimming speeds exceeded 25 cm/s. If such a stable orientation is maintained and compensate the low speeds during the day, the eels that leave during autumn and winter will be able to arrive at the spawning area during the next spring to summer.

It should also be noted that the swimming speeds in body length per second were significantly higher in shallow water during the night than in deep water during the day. In the open ocean, anguillid species exhibit DVMs during oceanic migration, swimming at depth during the day and in the shallows during the night^9–22^. These DVMs are likely related to the possible avoidance from visual predators under light conditions^19^ or maturation control^59^. Essentially, through the DVMs, the eels encounter low temperatures (< 10 °C) and high temperatures (> 20 °C) during the same day. Generally, the swimming speeds of fishes are restricted by the ambient water temperature^60^, and the water temperature encountered through DVMs might influence the horizontal-swimming speeds of Japanese eels.

Other factors besides swimming speed are important for the success of eel migrations, such as adapting to mesopelagic zones that silver eels undergo during their spawning migrations. The most important and obvious morphological adaptation in mesopelagic fish is their well-developed eyes, and migrating eels also seem to use this strategy. These fish often have relatively large pupils^61^, high photosensitive structures, such as tubular eyes^62^, a pure rod multibank retina^63^, and maximum rhodopsin absorption to adapt to the blue-green light in the deep sea^64^. The eyes of catadromous eels displayed enlargement during their transformation into migrating silver-phase eels^65, 66^ and potentially increase their retinal surface area, which results in the possibility of increased photon capture. In addition, the rhodopsins in the eyes change from a freshwater type with a maximum absorption of ~ 500 nm to a deep-sea type with a maximum absorption of ~ 480 nm^67–69^. Their extreme sensitivity to light is evident through their DVM in mesopelagic water, where the timing of a large descent and ascent in the DVM demonstrated by migrating catadromous eels is precisely synchronized with sunrise and sunset. Furthermore, eels alter their swimming depth in response to the phase of the Moon^9, 15, 20, 21^, appearing to be capable of perceiving extremely low-intensity moonlight.

This study showed that three eels released in the TS area (mainly 300–400-m depth) and one eel in the KC area (near surface) were found to change their swimming direction around the time of the solar culmination when the Sun’s bearing changed. The clockwise and counterclockwise trajectories of these eels corresponded to whether the Sun moved from the east to west in the southern and northern sky, suggesting that they demonstrated horizontal negative phototaxis swimming to avoid sunlight. They might move to avoid high-intensity sunlight horizontally, not vertically, as they gradually increase the swimming depths possibly due to acclimation to cold deep water after release. The daytime swimming depths of the eels became deeper day-by-day after their release (Fig. 4); a similar phenomenon was observed in European eels^12^, American eels^17^, and long fin eels^13^. Recently, Higuchi et al.^20^ observed that the daytime swimming depths of Japanese eels released in the TS area gradually became deeper until 13 days after their release. These facts indicate that they gradually acclimate to the cold water at the deep depths after release. Since this tracking study was conducted 2–8 days after their release, the daytime swimming depth of eels would not have reached a steady state yet. The relatively high intensity from sunlight at the shallow depths where eels swam immediately after release in the TS area might cause horizontal avoidance behavior from the light.

In other cases, many eels, especially those released in the KC area, did not demonstrate the rotational behavior. The eels in the KC area mostly stayed deeper (500–800 m) during the day than the eels in the TS area (stayed at depths of 300–600 m) even during the periods shortly after their release. This is possibly due to higher water temperatures even at the deeper depths in the KC area (Fig. 4). The eels in the TS area did not demonstrate clear rotational behavior at depths of more than 400 m. The PSAT studies have reported that the steady swimming depths during the day were 500–800 m^14, 20^. Therefore, it was assumed that the rotational behavior observed in some eels was not a regular behavior during their migration. However, the rotational behavior observed in this study suggests that they surely perceive the horizontal direction of Sun’s bearing at 400 m depths at least. Generally, they exhibit DVM precisely synchronizing with sunrise and sunset and surely perceive the change in sunlight intensity at deeper depths^9–22^. Even though the rotational behavior were not observed below 400 m, it remains unknown whether the eels could not perceive the Sun’s bearing from the light penetrated at depth; thus, further investigation of response to underwater light is required in future.

**Figure 6:**
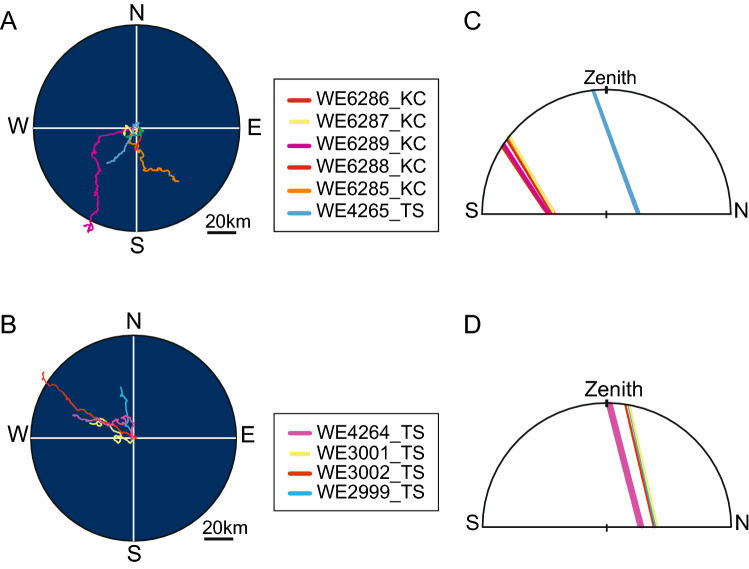
Swimming trajectories of eels and solar paths in the celestial sphere viewed from east during each tracking period. Swimming trajectories of eels released at (**A**) 20°N in the tropical–subtropical area and the Kuroshio Current area, and (**B**) 12°–14°15′N in the tropical–subtropical area. Solar paths through the north (N)–south (S) axis and the zenith at the time of tracking in (**C**) 20°N in the tropical–subtropical area and the Kuroshio Current area, and (**D**) 12°–14°15′N in the tropical–subtropical area.

While possible negative phototaxis behaviors were observed in some eels after release around the time of solar culmination, the trajectories of ten eels during the entire period of tracking experiments implied that each eel tended to swim meridionally toward the bearing of the Sun at culmination. We observed that eels released at middle (20°–34° N) and low (12°–13°N) latitudes tended to swim southward and northward in the meridional direction, respectively (Fig. 6A, B). The tendency to move in a north–south swimming direction corresponded to whether the Sun culminated to the north or south: eels swam southward if the culmination occurred in the southern sky, but they swam northward if it occurred in the northern sky (Fig. 6). In the KC area (33°–35° N), the Sun rose in the southeast, passed celestial meridian in the southern sky, and set in the southwest (Fig. 6C). At 20° N in the summer time when the tracking study was conducted, the Sun also passed a celestial meridian in the southern sky, but rose in the northeast and set in the northwest (Fig. 6C). When Sun culmination occurred in the southern sky, the meridional swimming directions tended to be southward (Fig. 6A). However, at 12° to 13° N in the summer time, the Sun rose in the northeast, passed the celestial meridian in the northern sky, and set in the northwest (Fig. 6D). When the Sun at culmination appeared in the northern sky, the meridional swimming directions tended to be northward (Fig. 6B). Furthermore, the swimming behavior by one eel (WE4264_TS) that was released slightly south (14° 15′ N) from the latitude with the Sun passing through the zenith was also indicative of the meridional swimming traits. This eel moved in a northerly direction on the first day, but then it lost its north–south bias in swimming around 14° 30′ N, where the Sun nearly passed through the zenith (Figs. 1 and 6D). These observations imply that the eels might move toward the latitude with the Sun passing through the zenith.

**Figure 7:**
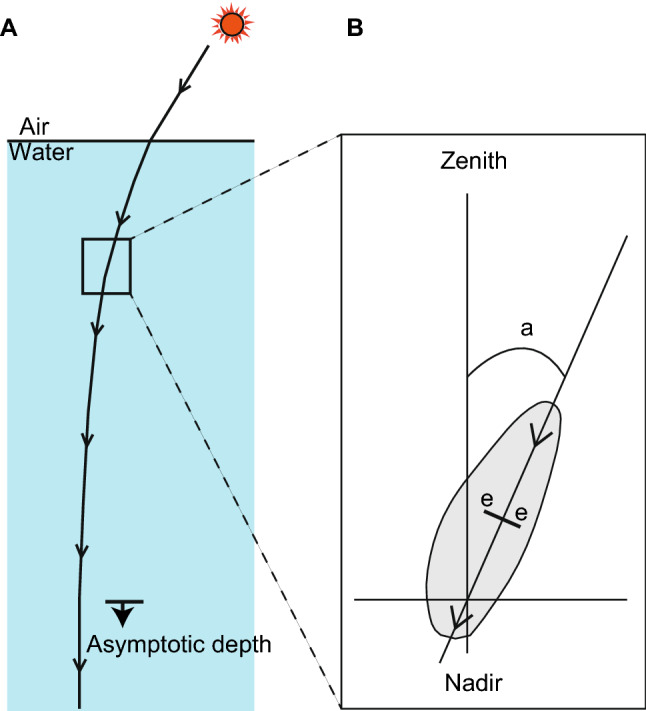
Optical features of underwater sunlight. (**A**) Schematic diagram of sunlight penetrating the deep ocean at 90° to the solar bearing. The line of arrows indicates the major axis of the incident beam in a vertical plane perpendicular to the Sun’s bearing. Blue light (around 475 nm) reaches the lowest depths. With increasing depth, the light field alters its character into a less directed distribution and a lower energetic level through scattering and absorption processes. Penetrating light rays are symmetrical around the region below the asymptotic depth. (**B**) An example of spectral radiance distribution (e. g. 475 nm) at a certain depth. The radiance distribution is shown by an ellipsoid and the major axis is drawn by a line with arrow. The refracted angular deviation (a) of the major axis of underwater radiance distribution from the vertical axis equals the tilt of the electric vector (ee bar) from the horizontal axis^53^. When the Sun’ s elevation was 60° in the Sargasso Sea, the radiance distributions were measured at three different depths and the tilt of the electric vector were estimated to be 24° at depths of 100 m and 200 m and 13° at depth of 400 m^52, 53^.

Theoretically, it is possible for mesopelagic animals to use solar cues for navigation at depths shallower than the asymptotic depth, below which penetrating light rays are symmetrical around the vertical axis and the polarization plane becomes horizontal. For example, the Sargasso Sea, where the two Atlantic catadromous eels spawn^1, 3^, has extremely transparent water^70^, and the major axis of radiance distribution still remains tilted in the mesopelagic zone. The angle of maximum radiance of sunlight at 475 nm was 13° at depths of 400 m when the Sun’s elevation was 60° (Fig. 7)^52, 53^. In highly transparent water, the asymptotic depth could be as high as 1000–1200 m, and the depths below this cannot be utilized for compass use^53^. Currently, it is not possible to verify whether the Sun culminating to north or south caused the meridional swimming tendencies of eels in this study. Potentially, these meridional swimming tendencies could be due to other orientation clues, such as the geomagnetic field, as discussed for temperate anguillid eels^17, 45^. Nevertheless, in future studies, it would be worthwhile considering solar cues as a possible candidate factor in the orientation of eels, even when under faint underwater light conditions.

Given that eels might be able to use the Sun’s bearing at culmination to orient their meridional swimming direction, this orientation scheme could support a clockwise eel migration route following a partial subtropical gyre^2, 37^. Japanese eels that departed from the nursery area first transported northeastward via the strong KC. Maintaining southward swimming in the current, they eventually crossed the current and shifted to the southward migration course. When they enter the KC, movement to the left of the bearing of the Sun at culmination (i.e., south) is the typical pattern for the early migration of eels from Japan. The movements of eels observed in the KC were consistent with the expected route; however, eels released at low latitudes of the TS area often swam northward but also westward, which resulted in their traveling an unreasonable distance from the spawning area. This might be due to their behavior during early migration. In this study, eels were transported from Japan and released into the open ocean at low latitudes. They might have swum toward the expected bearing of the Sun at culmination as if they were in the north and moved to the left of the Sun’s bearing along with the North Equatorial Current, which would mimic the early migration of eels leaving Japan and moving along the KC.

Among the eels tracked in this study were individuals with impaired swim bladders, yellow-phase eels in the process of hormone-treatment maturation, and silver-phase eels collected from different rivers in different years. Despite these variations, the swimming characteristics of the eels did not differ in terms of their DVM behavior^16^ and swimming speed. Nevertheless, confirmation of our results using samples with a uniform status in future research would be highly desirable. In this study, the tracked eel position was assumed to be identical to that of the tracking ship and the errors between these two positions could not be evaluated; thus, the positioning of tracked fish also may need to be improved in future studies. Experimental studies, such as tracking of blind, magnetically disturbed, or olfactory-blocked eels, could help obtain or eliminate alternative candidate clues and enhance our understanding of the navigational system of anguillid eels. Controlled laboratory experiments are required to directly quantify the ability of eels to perceive radiance distribution or polarization, along with any associated behaviors. In addition, the internal clock of eels required to perform celestial navigation should be investigated. Meanwhile, the results obtained from this study can enhance our knowledge of the mechanisms underlying the migratory behaviors of eels in the open ocean.

## Materials and methods

### Tracking experiment

The tracking data of Japanese eels from Chow et al.^16^ was used to analyze the horizontal-swimming behavior of eels in the open ocean. Wild eels were captured, commencing in the autumn before the tracking experiments (Table 1). Beginning 1 month before the research vessel departed for tracking in the TS area, six eels were injected weekly with salmon pituitary extract to induce maturity. Morphological measurements revealed that 8 of 11 eels were in the silver phase, whereas the remaining three eels were in the late yellow phase, according to an established classification that uses body colorations^66^ (Table 1). The late yellow-phase eels had an ocular index of 4.9–5.7, thus falling within the silver phase^66^; therefore, they appeared to be transitioning to silver eels.

Tracking was performed using depth-sensitive (P) and depth/temperature-sensitive (TP) ultrasonic transmitters (V16P and V16TP, VEMCO, Halifax, Canada) and the SEA TRACK VP170-PC (VEMCO) biotelemetry system installed on the R/V *Shoyo Maru*. A hydrophone with an array of 17 receiving elements was installed under the bottom of a ship, and each of 16 of these elements were directed to the eight points of the compass with a lean of 0° (horizontally) and 45° (slantwise); one element was directed with a lean of 90° (downward). The receiver recorded the depth and temperature data transmitted from the tracked fish every few seconds and estimated the vertical/horizontal directions from the vessel to the fish using the difference of the received signals among the elements. The horizontal distances between the fish and the vessel were estimated using the vertical directions from vessel and transmitted fish depths. The vertical/horizontal angular resolutions are both 11.25°. In actual situations, the estimation values of the fish position relative to the vessel fluctuate as the tracking vessel moves with waves. During tracking, the vessel was carefully operated to place the fish in front of the vessel’s bow with a constant horizontal distance of 200–400 m while referring to the estimated fish positions. Therefore, the positions of the tracking vessel were assumed to be identical to those of the fish, and time was expressed using the Japan Standard Time, i.e., JST, which is 9 h ahead of the Greenwich Mean Time, i.e., GMT.

Chow et al.^16^ reported that two yellow-phase eels (WE4264_TS and WE4265_TS) shared similar DVM patterns with silver eels and were thus considered behaviorally analogous to silver eels. However, a third yellow eel (WE4263_TS) did not exhibit DVM. The transmitters were surgically inserted into the abdominal cavities of large eels. However, as the specimens WE4263_TS (70.5 cm TL) and WE4265_TS (72.2 cm TL) were relatively small, the transmitters were externally attached to their backs. Chow et al.^16^ assumed that the weight or external attachment of transmitters might affect the swimming behavior of the small eel WE4263_TS; however, the effect of transmitter attachment varied by individual as the other eel WE4265_TS exhibited standard DVM patterns. Accordingly, the data of WE4263_TS that did not exhibit DVM were excluded from the following analysis of horizontal movements in this study. Further, during the study conducted by Chow et al.^16^, a 5 × 2-mm piece of the swim bladder was removed from three wild eels (WE6286_KC, WE6287_KC, and WE6288_KC) to investigate the effect of the organ on DVM. However, the resultant DVM profiles of these eels were generally comparable with those of untreated eels, except that one individual (WE6287_KC) exhibited a peculiar movement of swimming in a very shallow water in the first day. Chow et al.^16^ concluded that migrating eels do not depend on their swim bladder to maintain neutral buoyancy, specifically at depth.

### Data processing

The geographic position of the tracking vessel was recorded every minute using the Global Positioning System, and was assumed to be identical to the position of the tracked fish in this study. The travel vectors (including active swimming and transport by currents) of the eels were calculated using the geographic positions of the vessel at 10-min intervals. The horizontal vectors of the current were measured at a depth of 16-m layers from 32- to 816-m depths using an ADCP (Ocean Surveyor VM-ADCP, RD Instruments). The current vector of the water layer closest to the eel swimming depth was defined as the transport vector, and the transport vectors were averaged every 10 min. Active swimming vectors were obtained by subtracting the transport vectors from the eel travel vectors. The swimming speed when viewed in short time and effective swimming speeds over the tracking period of each eel were calculated from the swimming vectors: the former is the average magnitude of 10-min interval swimming vectors per second, whereas the latter is the magnitude of the mean vector of 10-min interval swimming vectors during all day and all night in the tracking, respectively. The initial nights of tracking were excluded from all analyses to avoid possible release-related effects. Day and night were indicated by sunrise and sunset. The travel distance on each day was defined as the distance traveled in 24 h from sunrise. Eels demonstrated upward swimming from 10 min before to 80 min after sunset and downward swimming from 60 min before to 30 min after sunrise^16^. The data collected during these periods was not used in our analysis of horizontal speeds to avoid interference from DVM factors. The altitude and azimuth of the Sun (see astronomic terminologies in Supplementary Figure 1) during the tracking experiments were obtained from the Naval Oceanography Portal (http://www.usno.navy.mil).

### Statistical analyses

The travel distances per day were compared between the TS and KC areas using Welch’s *t*-test. To investigate the possible effects of the time of day (day/night), developmental stage (yellow phase/silver phase), and swim bladder treatment (malfunctioned/intact) on their swimming speeds in body length per second (BL/s), the swimming speeds with Gaussian distributions were fitted to linear mixed models using the time of day and developmental stage in the TS area or using the time of day and swim bladder treatment in the KC area, respectively, as possible fixed effects and using the specimen ID as a random effect. The swimming speeds in body length per second were calculated from the swimming speeds at 10-min intervals divided by body lengths.

The mean and standard deviation of the magnitude and bearing of each vector were calculated to summarize the travel, transport, and swimming vector obtained at 10-min intervals for individual fish. Here, the standard deviation of bearings, *S*, was defined as $S = \sqrt{-2log(R)},$ where *R* indicates the mean resultant length of circular data. The uniformity of the circular distribution on the swimming vectors of each eel during day and night was tested using the Rayleigh test.

To determine whether eels released at various locations could swim toward their spawning area (12–15° N in latitude, 140.5–143° E in longitude) in the meridional (north–south) and zonal (east–west) axes, all 10-min interval swimming vectors were categorized as northward–southward and eastward–westward. In the meridional analysis, the swimming vectors with angles of 0°–90° and 270°–360° were categorized as northward vectors and 90°–270°° as southward vectors. In the zonal analysis, the swimming vectors with angles of 0°–180° were categorized as eastward vectors and 180°–360° as westward vectors. The uniformity of the frequency of these vectors in each eel was tested using the chi-squared test, compared with a uniform expected frequency under the null hypothesis. In any test, the level of statistical significance was set to 0.05. Statistical analyses were conducted in R version 3.6.0^71^ using the ‘lme4’ (ver. 1.1–21), ‘lmerTest’ (ver. 3.1–0), and ‘circular’ (ver. 0.4-93) packages.

### Ethics

At the time of sampling (September 2009 and November 2012), *A. japonica* was not an endangered species. Wild eels were purchased from local fishers with fishing licenses issued by fisher cooperative associations of the Tone River (Chiba Prefecture), Amikake River (Kagoshima Prefecture), and Tokyo Metropolitan Government (the Naka River). Our experiments were conducted in accordance with the institutional guide of the National Research Institute of Fisheries Science, Japan Fisheries Research and Education Agency.

## Supplementary Information

Supplementary Information.
