# Supplementary material for: Active swimming and transport by currents observed in Japanese eels (Anguilla japonica) acoustically tracked in the western North Pacific
Source: Sci Rep. 2022 Mar 1;12:3490. doi: 10.1038/s41598-022-05880-x (PMC8888653; doi:10.1038/s41598-022-05880-x)
Supplement: Supplementary file 1 — Supplementary Information. [file 41598_2022_5880_MOESM1_ESM.pdf]

Supplementary material for "Active swimming and transport by currents observed in Japanese eels (*Anguilla japonica*) acoustically tracked in the western North Pacific"

Nobuto Fukuda[a],*, Toshihiro Yamamoto[a], Kazuki Yokouchi[a], Hiroaki Kurogi[a], Makoto Okazaki[a], Yoichi Miyake[b], Tomowo Watanabe[a], Seinen Chow[a],*

[a]Yokohama station, Japan Fisheries Research and Education Agency, 2-12-4 Fukuura, Kanazawa, Yokohama, Kanagawa 236-8648, Japan; [b]Hatsukaichi station, Japan Fisheries Research and Education Agency, 2-17-5 Maruishi, Hatsukaichi, Hiroshima 739-0452, Japan
*These authors equally contributed to this work.

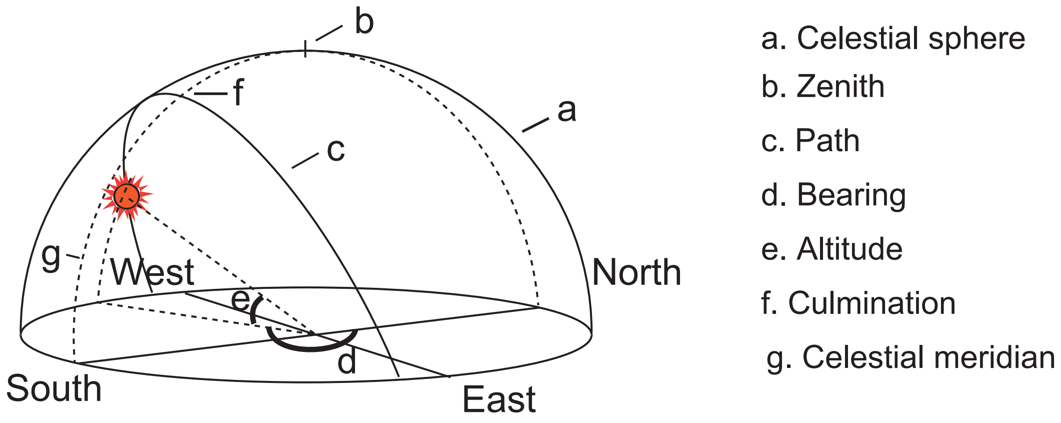

Supplementary Fig. 1 Astronomic terminologies
a. Celestial sphere is an imaginary sphere surrounding the Earth and is centred at the observer position upon which all celestial bodies can be projected. b. Zenith is the point on the celestial sphere vertically above an observer's position. c. Path is the trajectory that a certain celestial body (the Sun drawn in the figure) adheres to at a certain time in the celestial sphere. d. Bearing is horizontal angular measurement from true north (north = 0 degrees, east = 90 degrees, south = 180 degrees, west = 270 degrees). e. Altitude is an angle of a certain celestial body relative to the Earth's horizon. f. Culmination is the time when a celestial body reaches the observer's meridian. g. The celestial meridian is the line passing through the celestial poles and the zenith.
